# Economic potential of essential oil production from New Zealand-grown *Eucalyptus bosistoana*

**DOI:** 10.1038/s41598-023-40632-5

**Published:** 2023-08-28

**Authors:** Chamira Rajapaksha, Paul Greaves, Clemens M. Altaner

**Affiliations:** 1 School of Forestry | Kura Ngahere, University of Canterbury, Christchurch, New Zealand; 2 UNWUW Limited Trading as Estate Aromatics, PO Box 95, Riversdale, Southland 9776 New Zealand

**Keywords:** Plant sciences, Environmental social sciences

## Abstract

Farm foresters and other growers are establishing a ground-durable hardwood resource, including the emerging plantation species *Eucalyptus bosistoana* in New Zealand. The foliage of this species contains essential oils in quantity and quality suitable for commercial extraction. Essential oil production could improve the economic viability of *E. bosistoana* plantations, diversifying the grower’s income and providing an early revenue stream. This study assessed the economic potential for essential oil production from New Zealand grown *E. bosistoana* plantations. A sensitivity analysis indicated that uncertainty of leaf biomass availability, genetic as well as seasonal changes in oil content, and fluctuations in essential oil price are equally important on the viability of an essential oil operation. Small-scale essential oil production could be sustainably supplied with foliage from thinning and pruning operations sourced from the envisaged regional planting programmes and commence in 3–5 years. A large-scale operation could be supplied when trees will be harvested. Lastly, based on the operational costs of a domestic small-scale essential oil producer, oil value from *E. bosistoana* would exceed the cost of production.

## Introduction

*Eucalyptus bosistoana* is an emerging plantation species in New Zealand and improved planting stock is commercially available since 2020^1^. It is sustainably grown to supply class 1 ground-durable hardwood^2^ for substituting unsustainably harvested tropical hardwood imports and preservative treated pine^3^. It has potential to be used in high stiffness engineered wood products. While there is interest of larger forest grower cooperations, farmers and vineyard owners are establishing smaller plantings. These plantings must be concentrated in local catchments to ensure a future market for the ground-durable hardwood by being able to sustainably supply a wood processing facility. The New Zealand Dryland Forest Initiative (NZDFI) promotes the establishment of up to ten durable eucalypts catchments of 5000 ha within 40 km of an identified wood processing site between 2020 and 2050^4^.

Apart from its valuable timber, the foliage of *E. bosistoana* was reported to contain essential oils which are comparable in quantity and quality to *E. globulus*, the main source of eucalyptus essential oil^5–8^. While small-scale essential oil producers exist in New Zealand, no eucalyptus essential oil is produced. Eucalyptus essential oil production is often part of small-scale dual-purpose eucalyptus plantations able to support the grower with an early revenue stream^6,9^. In such silvicultural regimes, foliage for essential oil production is available from pruning, thinning and harvesting operations.

Leaves from pruning *E. smithii* and *E. dives* timber plantations have been used for essential oil production in China. The same procedure was practiced in pulpwood and firewood plantations of *E. citriodora*, *E. globulus* and *E. camaldulensis* in India. Pruned leaves of *E. citriodora* which have been planted for charcoal production have also been used for perfume industry oil production in Brazil^6^. Pruning operations were reported to yield 6–7 kg of fresh leaf biomass per tree for *E. grandis*^10^ and 1.5–3.5 kg for *E. citriodora*^6,11^.

Thinning operations can be classed into ‘thinning to waste’ or ‘production thinning’. In contrast to ‘thinning to waste’ where the trees including their foliage are left in the stand, ‘production thinning’ extracts the stems for timber production and therefore offers the opportunity to obtain foliage in the same operation. Costs for ‘production thinning’ are higher in steep terrain and not necessarily matched by the timber value^12^. Realising additional revenue from the waste leaves could make such operations more profitable.

Essential oil yield of a eucalyptus plantation is best expressed as oil yield per area per time. It has been reported that the essential oil yield of a species is controlled by the available leaf biomass and the oil content of the leaves^6,13–15^. Therefore, in addition to the variation of leaf oil content, the leaf biomass per unit area of the plantation and the growth rate of the trees need to be considered when estimating economics of essential oil production of *E. bosistoana*.

*E. bosistoana* is an emerging plantation species and no data on oil yields or leaf mass have been reported. To estimate oil yields for such plantations, growth and allometric functions for eucalypts were reviewed, applied to proposed silvicultural regimes for *E. bosistoana* in New Zealand, and compared to a limited number of measured foliage masses. Combined with collated information on oil yields in *E. bosistoana* foliage, eucalyptus essential oil price and distilling operations, the economic potential of essential oil production for *E. bosistoana* plantations was estimated.

## Methodology

### Foliage moisture content

Three fresh *E. bosistoana* leaf samples of ~ 20.0 g were collected from unknown progeny and dried to constant weight at 60 °C. The collection and handling of plant material was in accordance with the relevant guidelines. Moisture content (MC) was expressed as mass of water in relation to the fresh weight,

$$MC \left( \% \right) = \frac{{W_{f} - W_{d} }}{{W_{f} }} \times 100$$

where ${W}_{f}$ is the leaf fresh weight and ${W}_{d}$ is the leaf dry weight.

The moisture content of fresh *E. bosistoana* leaves was 48.2%. This value fell into the reported ranges for *E. miniata*, *E. tetrodonta* and *E. papuana* (31–59%, 47–57% and 34–40%, respectively)^16^ and *E. regnans* (49.5–58.6%)^17^. As leaf moisture contents were reported to vary seasonally^17,18^, an average moisture content ~ 50% for eucalyptus leaves appears to be a sensible estimate.

### *E. bosistoana* leaf biomass measurements

Eight 7 years old *E. bosistoana* trees of unknown progeny grown in Christchurch, New Zealand were assessed for young and mature leaf biomasses (Table 1). These trees had a mean fresh leaf weight of 5.50 kg at a mean diameter at breast height (DBH) of 6.8 cm, ranging from 3.45 kg (at 5.1 cm DBH) to 10.95 kg (at 7.7 cm DBH).

**Table 1:** Leaf biomass and DBH of 7 years old *E. bosistoana.*

Tree No	DBH (cm)	Fresh leaf biomass (kg)		Total fresh leaf biomass (kg)
		Young	Mature	
1	6.0	0.70	3.30	4.00
2	7.1	0.85	4.40	5.25
3	7.2	0.10	4.30	4.40
4	7.7	1.55	9.40	10.95
5	6.8	1.10	3.30	4.40
6	6.7	0.80	3.30	4.10
7	5.1	1.45	2.00	3.45
8	8.0	2.10	5.60	7.70
**Mean**	**6.8**	**1.08**	**4.45**	**5.50**

### Literature review of leaf biomass models for eucalypts

Published allometric regression models for leaf biomass of different eucalyptus species were collated (Table 2). As models refer either to fresh or dry weights, dry weights were converted to fresh weights using the MC determined for *E. bosistoana* leaves.

**Table 2:** Allometric regression models for foliage biomass of eucalyptus species including the number of trees assessed in the studies and the represented range of diameter at breast height (DBH).

Species	Regression equation	No. of trees	DBH range (cm)	References
*E. globulus*	W = 0.09 × D^1.9^ (dry)	83	1–20	^19^
	W = 0.0287 × D^1.9002^ (dry)	230	2–35	^20^
*E. tetrodonta*	W = 0.046 × 0.2068 × D^2.3191^ (dry)	14	5–40	^16^
	W = 0.001 × D^3.03^ (fresh)	8	5–30	^21^
*E. miniata*	W = 0.038 × 0.1527 × D^2.390^ (dry)	14	5–40	^16^
	W = 0.003 × D^2.44^ (fresh)	8	5–30	^21^
*E. papuana*	W = 0.029 × 0.0356 × D^2.8567^ (dry)	12	5–40	^16^
*E. bleeseri*	W = 0.01 × D^1.91^ (fresh)	8	5–30	^21^
*E. porrecta*	W = 0.007 × D^2.21^ (fresh)	8	5–30	^21^
*E. nitens*	lnW = (− 12.060 + 2.1307) × ln(D^2^) (dry)	27	20–40	^22^
*E. crebra*	lnW = (− 5.785 + 1.858) × ln(C) (fresh)	20	2–40	^23^
*E. melanophloia*	lnW = (− 6.227 + 1.851) × ln(C) (fresh)	20	2–40	^23^
*E. populnea*	lnW = (− 3.491 + 1.259) × ln(C) (fresh)	22	2–40	^23^

W: leaf weight (kg), D: diameter at breast height (cm), C: circumference (cm), ln: natural logarithm.

## Results and discussion

### Leaf biomass

**Figure 1:**
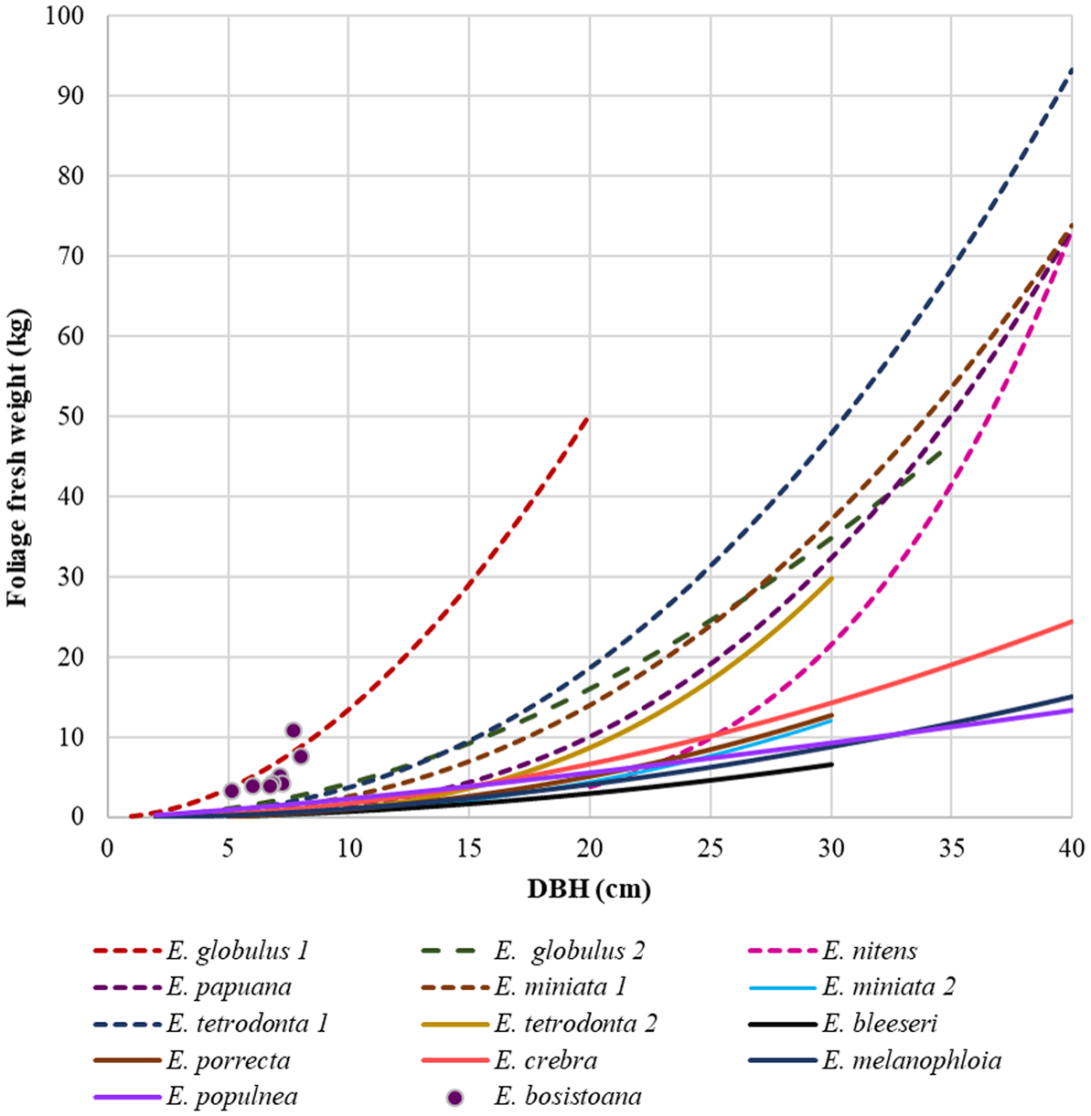
Foliage fresh weights depending on DBH of different eucalyptus species. Solid lines show models developed for fresh weights. Dashed lines indicate the models developed for dry weights and subsequently converted to fresh weights using a MC of 48.2%. Models are plotted for the DBH range represented in the respective data. Leaf biomass measured for eight 7 years old *E. bosistoana* trees are represented by dots.

Published models (Table 2) for fresh weights of leaf biomass of eucalyptus species depend on DBH as illustrated in Fig. 1. The model curves were restricted to the DBH ranges represented by the trees underpinning the models. Models converted from dry mass appeared to predict higher fresh leaf masses than models based on fresh leaf measurements (Fig. 1). However, overlapping variation was still observable between foliar biomass models based on dry or fresh measurements, respectively.

The exact relationship between DBH and foliage mass is unknown for *E. bosistoana*. The available *E. bosistoana* leaf biomass data were at the upper end of the scale and best predicted by the *E. globulus* models (Fig. 1). More data, in particular for larger trees and different silvicultural regimes, is needed to develop leaf biomass models for *E. bosistoana*.

### Sensitivity analysis of factors affecting *E. bosistoana* essential oil production

#### Foliage biomass

The average of the *E. globulus* 1 and 2 models was used to predict foliage mass for *E. bosistoana* from DBH, as these seemed to best fit the few available data (Fig. 1), with the individual *E. globulus* models used as best and worst case. The *E. globulus* studies were also those based on the largest data sets (83 and 230 trees, respectively) and therefore more trustworthy than the others based on less than 30 trees at best (Table 2). The best and worst leaf mass scenarios differed by 52% from the average (Table 3).

#### Seasonal variation of oil content in leaves

Seasonal changes in volatile leaf extracts have been reported for other eucalyptus species^24,25^ and aromatic herbs^26^. *E. bosistoana* oil yields vary seasonally, and highest and lowest yields were obtained in summer and winter, respectively^27^. Oil yield per tree can be increased by 75% when harvesting only in summer compared to harvesting throughout the year. On the other hand, oil yield could decrease by 53% when harvesting only in winter (Table 3). While seasonality of essential oil production is inevitable for annual herbs, continuous production is possible for long-lived evergreen eucalypts. However, if production occurs throughout the whole year only the average annual yield and composition of the extracts can be achieved, with product quality varying throughout the year.

**Table 3:** Variation of leaf mass, leaf oil content and prices of best and worst cases compared to the average.

Criteria		Best	Average	Worst	Variation %
Fresh leaf biomass (kg/tree)	At 30 cm DBH	109.0	71.9	34.8	± 52%
Oil content (mg/g fresh leaf)	Seasonal	17.7	10.1	4.7	+ 75% (best) − 53% (worst)
	Genetic	21.2	13.3	7.5	+ 59% (best) − 44% (worst)
Oil price^a^ (NZD/kg)	Conventional	66	45	24	± 47%
	Organic	110	88	66	± 25%

^a^Oil prices were converted from USD to NZD (1 NZD = 0.68 USD).

#### Genetic variation of oil content in leaves

Oil content of *E. bosistoana* was shown to be under genetic control (heritability *h*^*2*^ = 0.25) with the best family performing 59% above the average, while the worst performing family was 44% below the average^27,28^. However, it needs to be kept in mind, that foliage mass has an overriding effect on the oil yield of a plantation and therefore, oil yield should be considered on a plantation area basis rather than per leaf mass^6^. Since terpene biosynthesis pathways in leaves, tree growth rate, DBH, crown density and canopy size are under genetic control, it has been shown for eucalypts that choosing the right family from breeding programs is still useful^29–31^. However, biomass correlated traits have lower heritability than oil traits^32^. Shorter harvesting cycles can be implemented if the genotypes are fast growing. High essential oil yielding genotypes have increased growth rate, leaf biomass as well as oil quality. And relevant to dedicated short-rotation eucalyptus essential oil plantations, the ability to coppice is also controlled by genetics^33^. The Australian eucalyptus essential oil industry is based on *E. polybractea* and relies on a breeding programme to increase productivity^34^.

Apart from oil yield, oil quality, i.e. the 1,8-cineol content, is also under genetic control^28^. In contrast to oil yield, selection of genotypes which yield oil of superior quality is not directly affected by leaf biomass.

#### Essential oil price

Eucalyptus essential oil prices in the global market were fluctuating over time and depend on supplying country, oil type (species) and grade. Prices were low in the early 1990s but have increased gradually over the last two decades. According to export data from China to Europe, prices rose from 3.5 to 12 USD/kg (5–18 NZD/kg) from 1997 to 2013^35^. Although China is the largest eucalyptus essential oil supplier in the market, the price for this product was low compared to oils produced in Australia, Portugal, Spain, India or Brazil^36^. In 2016 global market prices for essential eucalypt oils ranged from 16 to 44 USD/kg (24–66 NZD/kg)^37^. Fluctuations in conventional oil price were ± 47% of the average (Table 3). A premium is paid for organic oil, reaching from 45 to 75 USD/kg (66–110 NZD/kg). The price for higher grade *E. polybractea* organic oil was 135 USD/kg (199 NZD/kg)^38^. Organic oil value was roughly double that of the conventional oil value and appeared to be more stable (± 25%). The average price of eucalyptus oil in New Zealand was reported to be around 30 NZD/kg in 2021.

All considered variables appear to have comparable uncertainty on the predictions of eucalyptus essential oil yields (Table 3). However, seasonal variations^27^ are linked to production timing, i.e. cannot be exploited in a year-round production. In a seasonal production scenario, silvicultural operations generating waste leaves would best coincide with high essential oil contents. Selecting genetically superior planting stock would allow to increase the quality and quantity of oil in the foliage, but to increase oil yield of a tree or a plantation, growth is also important^28^. Considering oil price, an organic product had a more stable and higher value than conventional oil, however, it is unclear if and how timber plantations can be certified as organic.

### Distil capacity

**Figure 2:**
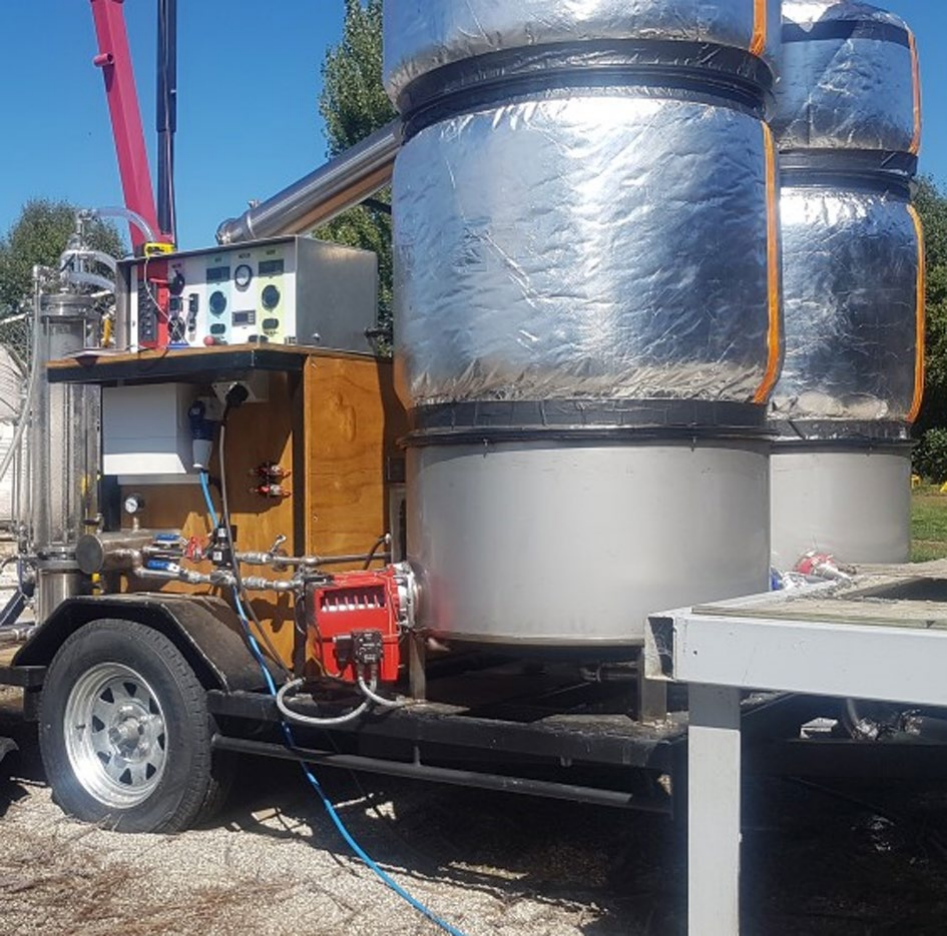
A mobile steam vacuum distil used for essential oil production in New Zealand.

Small-scale mobile distillery units are used in New Zealand for essential oil production. Such mobile units cost from 65,000 to 120,000 NZD depending on capacity, extraction method and efficiency. The mobile steam vacuum distil shown in Fig. 2 has a capacity for 200 kg raw material per run and costs 65,000 NZD. Without loading and unloading of leaf material, a distillation cycle for eucalyptus essential oil takes approximately 40 min. Yields of 2 to 4 kg of pure essential oil were reported from one cycle for some NZ grown eucalypts, i.e. 200 kg foliage. Mechanised foliage harvesting, as practised with mallees in Australia grown in dedicated essential oil plantations, allows larger operations and reduce labour costs. In this system, still boxes with a capacity of 1 to 4 t of fresh foliage are attached to a harvester and filled in the field.

Annual oil production is dependent on still capacity, still number, distillation runs per day and the number of working days in a year^39^. For example, in an *E. polybractea* operation one person needs about 1 h to fill a 3 t still box with fresh leaves and distillation takes another 1 h. One hectare of *E. polybractea* plantation can fill 2–2.5 stills. Typical oil yields of 40–60 kg for a load of 2.5 t fresh eucalyptus leaves were reported^6^. The extracted leaves can be used as mulch.

### Potential for eucalyptus oil production from *E. bosistoana* plantations in New Zealand

Information of a silvicultural regime is needed to calculate oil yields per area or the needed plantation area to supply a distil. Proposed plantation regimes for *E. bosistoana* plantations in New Zealand are for peeler/pole production (15–20 years) and sawlog production (30–40 years)^3^. For peeler/pole production, trees are planted at an initial stocking of 1,100 stems/ha and thinned to 600–800 stems/ha after 4–5 years when the trees have reached a DBH of 8 cm. A target diameter at full rotation of 30 cm is achievable in 20 years on suitable sites. A sawlog plantation with an initial stocking of 1000–1200 stems/ha will be reduced to 300–400 stems/ha after 4–5 years by thinning (DBH 8 cm) and harvested after 30 years with an average stem diameter of 45 cm or above (Table 4)^3^.

**Table 4:** Estimation of fresh leaf biomass, oil yield and oil value obtainable from *E. bosistoana* plantation regimes in New Zealand.

	Stems/ha	Fresh leaf biomass per tree (kg/stem)	Fresh leaf biomass^c^ (kg/ha)	Oil yield^d^ (kg/ha)	Oil value^e^ (NZD/ha)
Peeler/pole plantation; 20 year rotation on suitable site					
Planting	1100				
Thinning at DBH 8 cm	400	6.0^a^	2400	24.2	727
Pruning	700	4.0^b^	2800	28.3	848
Harvest at DBH 30 cm	700	72.0^a^	50,400	509	15,282
**Total**^f^	–	–	**55,600**	**561.6**	**16,848**
Sawlog plantation; 30 year rotation on suitable site					
Planting	1100				
Thinning at DBH 8 cm	750	6.0^a^	4500	45.5	1363
Pruning	350	4.0^b^	1400	14	423
Harvest at DBH 45 cm	350	155^a^	54,250	548	16,440
**Total**^f^	–	–	**60,150**	**608**	**18,227**

^a^According to average leaf biomass from the allometric model (Fig. 1).

^b^Assumed average of literature reports of pruning biomass^6, 10,11^.

^c^Stocking (trees/ha) * fresh leaf biomass per tree (kg/tree).

^d^Using the average annual oil yield of 10.1 mg/g^7^.

^e^Using the eucalyptus oil price of 30 NZD/kg in the 2021 New Zealand market.

^f^Total at the end of rotation.

Annual oil yields of 562 and 607 kg/ha/year were calculated for the peeler/pole and sawlog regimes, respectively (Table 4), using the average allometric scenario and oil yield (10.1 mg/g (fresh))^27,28^. These values were comparable to reports of annual oil yields from *E. globulus* plantations ranging between 450 and 900 kg/ha/year^6^. As both regimes generated similar amounts of leaves, the predicted oil value extractable per hectare from these plantations was with 16,860 NZD/ha and 18,210 NZD/ha also similar. As the size of the trees at harvest age differed, essential oil could increase the value of a tree by ~ 20 NZD in a peeler/pole regime and ~ 50 NZD in a sawlog regime utilising the assumptions of the base scenario.

Thinning and pruning operations combined generate only ~ 10% of the foliage biomass available from harvesting at rotation age. Sourcing leaves at harvest not only provides most of the material but is also likely the most cost effective, as foliage could be gathered more easily mechanically. Moving and handling of biomass from pruning or ‘thinning-to-waste’ operations is more difficult and will yield less product per hectare. Production thinning of sawlog plantation at a later age might be worth considering if whole trees can be extracted, as costs would be primarily covered by the timber value and adding value by utilising the leaves for oil production could improve the economic viability of such operations.

The required plantation area and value of oil production for a small-scale and a large-scale operation have been estimated (Table 5). The small-scale operation would require access to ~ 100–150 ha of *E. bosistoana* per year if it utilises thinning or pruning residues. These resources would be available 4–5 years after planting and equate to a total plantation estate of 3000–4000 ha. When utilising harvesting residues at rotation age only ~ 10 ha per year are needed to supply a small-scale distil. While the associate plantation estate would only be 150–250 ha, residues would not be available for 20–30 years after planting. The required plantation area would be 5 times as large for the large-scale eucalyptus oil operation. In other terms, a small-scale distillery requires about 20–25 30 cm DBH trees per day while a large distillery requires 100–125 trees per day.

**Table 5:** Estimation of plantation area required for small and large-scale oil production form *E. bosistoana* plantation regimes.

Regime	Foliage source	Plantation area (ha/year)^d^	Total plantation area (ha)
Small-scale operation^a^ Leaf biomass requirement^c^: 400 t/year Oil value^e^: 121,200 NZD/a			
Peeler/pole (20 year rotation)	Final harvest	7.9	158
	Thinning	167	3340
	Pruning	143	2860
Sawlog (30 year rotation)	Final harvest	7.4	222
	Thinning	89	2670
	Pruning	286	8580
Large-scale operation^b^ Leaf biomass requirement^c^: 2000 t/year Oil value^e^: 606,000 NZD/a			
Peeler/pole (20 year rotation)	Final harvest	39.5	790
	Thinning	833	16,660
	Pruning	715	14,300
Sawlog (30 year rotation)	Final harvest	37	1110
	Thinning	445	13,350
	Pruning	1430	42,900

^a^Using a 200 kg capacity distil operating eight cycles per day.

^b^Using two 2000 kg capacity distils operating two cycles per day.

^c^Assuming 250 working days per year.

^d^Leaf biomass requirement per year / leaf biomass per hectare (Table 3).

^e^Plantation area (ha) per year * oil value (NZD) per hectare (Table 3).

Considering NZDFI’s target of establishing ten 5000 ha catchments of durable eucalyptus plantations^40^, each of these would be able to support the modelled eucalyptus oil operations. As establishment of durable eucalyptus plantation has commenced in 2021, establishing an associated essential oil business could start soon at small-scale utilising thinning and pruning residues and grow into a larger-scale operation utilising harvest residues when the planation estate has matured.

### Economics of essential oil production form *E. bosistoana*

It should be noted that, since there is no commercial eucalyptus essential oil production in New Zealand, assumptions cannot be verified. Detailed costings of medium scale eucalyptus essential oil production facilities for developing countries including the establishment of associated dedicated plantations have been reported in 1992^39^. These are not necessarily transferable into the current New Zealand context.

A mobile small-scale *E. bosistoana* essential oil production facility, requiring a single operator and utilising foliage from thinning a timber plantation, was costed (Table 6). Operational costs for the distil were based on an existing domestic essential oil operation. As no data on production thinning costs of *E. bosistoana* plantations was available, costs for gathering foliage were estimated as follows: According to Taylor and Visser^12^, average production thinning operational costs for *P. radiata* plantations in New Zealand including the machine operating wages for tree felling, harvesting, loading and transporting were 4,210 NZD per day. Recovering 125 t of stems, this equates to 34 NZD per tonne. A firewood operation of Australian durable eucalyptus plantations was reported to be around 60 AUD per tonne (65 NZD/t)^41^. Assuming a green density of 1,000 kg/m^3^ a *E. bosistoana* stem with a DBH of 8 cm would weigh ~ 25 kg^42^, equating to 40 trees per tonne. Combining the extraction costs per tonne and the number of trees per tonne allowed to calculate extractions costs of 0.85 to 1.60 NZD per tree. According to the allometric model displayed in Fig. 1, such an *E. bosistoana* tree at 8 cm DBH has a foliage mass of 6 kg. Consequently, extraction costs per kg of leaf material from production thinning was estimated to be 0.14 to 0.26 NZD per kg of leaf material. The average cost of 0.20 NZD per kg of leaves were used for further calculations. Higher harvesting cost were published 30 years ago for dedicated *E. smithii* oil plantations in southern Africa: 1.25–1.91 USD/kg with, and 0.25–0.86 USD/kg without depreciation for machinery, respectively^39^.

An annual profit of 40,900 NZD/a was estimated, requiring an investment of 65,000 NZD for the distil (Table 6). Costs were dominated (60%) by gathering foliage. These costs could reduce if they would be partially covered by the production thinning operation, i.e. the timber revenue. Further, thinning costs were based on productions thinning costs, likely underestimating costs for the smaller 8 cm DBH trees used in this study. It also has been shown that choosing good genetics is vital for an independent producer establishing dedicated plantation for oil production under a short rotation coppice system^14, 43^.

**Table 6:** Costing of a small-scale *E. bosistoana* essential oil operation based on.

Revenue	
Assumed oil yield per day	3 kg/ch × 8 ch/d = 24 kg/d
Oil price	30 NZD/kg
Total earnings per day^a^	24 kg/d × 30 NZD/kg = 720 NZD/d
Costs	
Distilled leaf biomass per day	200 kg/ch × 8 ch/d = 1600 kg/d
Foliage per day^b^	0.20 NZD/kg × 1600 kg/d = 320 NZD
Labour cost for one person per day^c^	20 NZD/h × 8 h/d = 160 NZD/d
Diesel cost for extraction per day^d^	3.5 L/ch × 8 ch/d × 1.8 NZD/L = 50.4 NZD/d
Total cost per day	320 + 160 + 50.4 NZD/d = 530.4 NZD/d

Profit	
Operational profit per day	720 − 530.4. NZD/d = 189.6 NZD/d
Operational profit per year (250 days)	189.6 NZD/d × 250 d/a = 47,400 NZD/a
Depreciation distil (10 years)	65,000 NZD/10 a = 6,500 NZD/a
Overall profit per year	${47},{4}00 - {65}00\;{\text{NZD}}/{\text{a }} = { 4}0,{ 9}00\;{\text{NZD}}/{\text{a}}$

^a^Using 2021 the New Zealand market eucalyptus oil price of 30 NZD/kg.

^b^Using average production thinning cost as discussed in the text.

^c^Using New Zealand labour wage per hour^44^.

^d^^45^.

## Conclusion

Based on the available information, essential oil production from the emerging *E. bosistoana* planation estate can be commercially viable, supporting the financial viability of such plantations. The current planting programme will be able to sustain the supply of small-scale mobile distils with foliage in the next 3–5 years from pruning and thinning operations. The established essential oil producers utilising such mobile distils in New Zealand offer market entry for a *E. bosistoana* based essential eucalyptus oil. Sufficient foliage for large-scale essential oil production will be available in 20–30 years once harvest commences.

## Data Availability

The datasets used and/or analysed during the current study available from the corresponding author on reasonable request.
